# The Control of Cortical Folding: Multiple Mechanisms, Multiple Models

**DOI:** 10.1177/10738584231190839

**Published:** 2023-08-24

**Authors:** Alexandra Moffat, Carol Schuurmans

**Affiliations:** 1 Sunnybrook Research Institute, Biological Sciences Platform, Toronto, ON, Canada; 2 Department of Laboratory Medicine and Pathobiology, University of Toronto, Toronto, ON, Canada; 3 Department of Biochemistry, University of Toronto, Toronto, ON, Canada

**Keywords:** cortical development, cortical folding, gyrencephaly, lissencephaly, rodent models, nonhuman primate models, human disorders, extracellular matrix, signaling pathways

## Abstract

The cerebral cortex develops through a carefully conscripted series of cellular and molecular events that culminate in the production of highly specialized neuronal and glial cells. During development, cortical neurons and glia acquire a precise cellular arrangement and architecture to support higher-order cognitive functioning. Decades of study using rodent models, naturally gyrencephalic animal models, human pathology specimens, and, recently, human cerebral organoids, reveal that rodents recapitulate some but not all the cellular and molecular features of human cortices. Whereas rodent cortices are smooth-surfaced or lissencephalic, larger mammals, including humans and nonhuman primates, have highly folded/gyrencephalic cortices that accommodate an expansion in neuronal mass and increase in surface area. Several genes have evolved to drive cortical gyrification, arising from gene duplications or de novo origins, or by alterations to the structure/function of ancestral genes or their gene regulatory regions. Primary cortical folds arise in stereotypical locations, prefigured by a molecular “blueprint” that is set up by several signaling pathways (e.g., Notch, Fgf, Wnt, PI3K, Shh) and influenced by the extracellular matrix. Mutations that affect neural progenitor cell proliferation and/or neurogenesis, predominantly of upper-layer neurons, perturb cortical gyrification. Below we review the molecular drivers of cortical folding and their roles in disease.

## An Overview of Cortical Development at the Cellular Level

### A Primer on Cortical Structure

**Figure 1.**
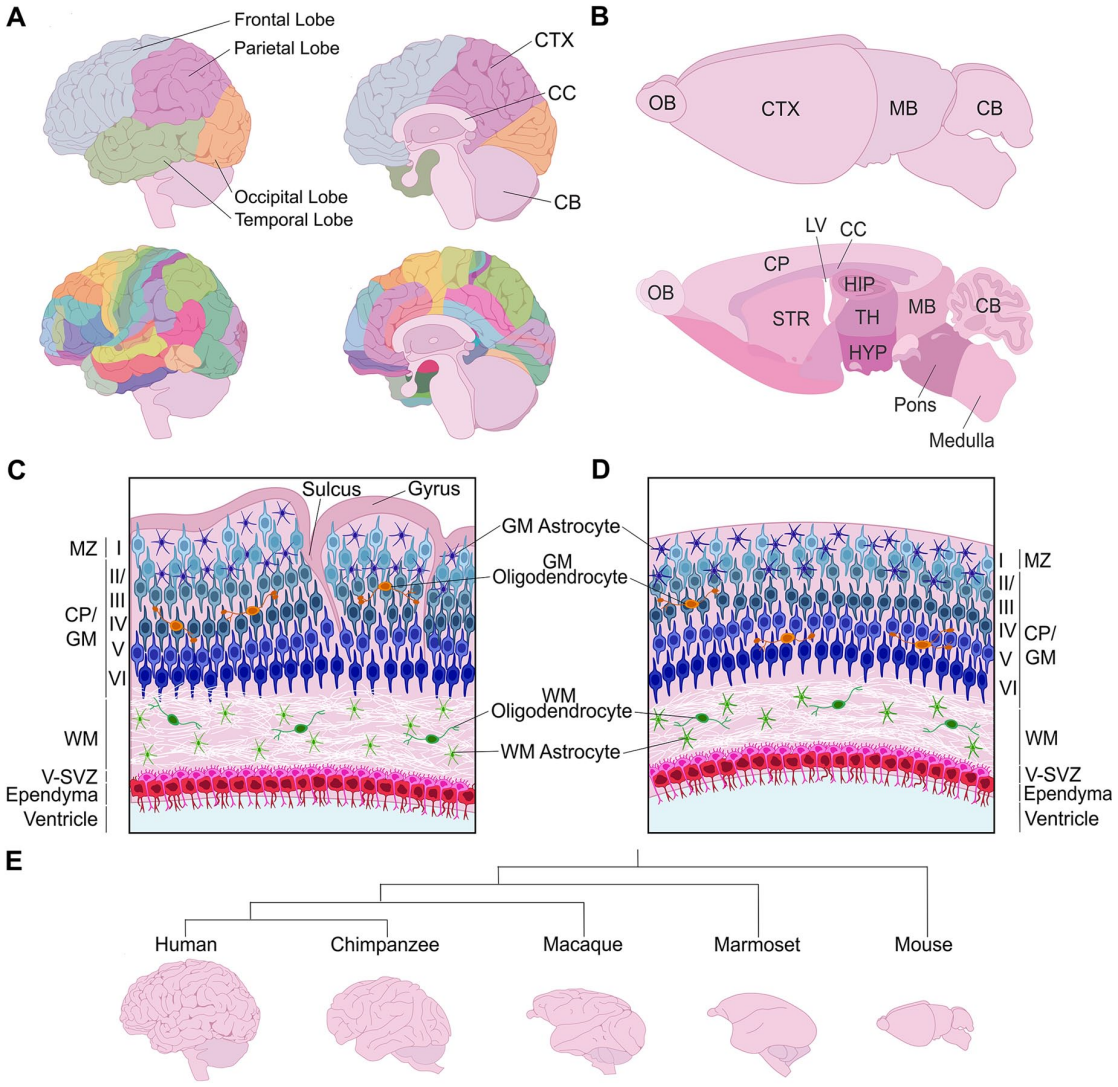
Structure of the mature cerebral cortex in gyrencephalic and lissencephalic species. (A) Illustration of a human brain, showing the four cortical lobes and Brodmann areas. (B) Illustration of the murine brain, highlighting the different brain areas in a sagittal section. (C, D) Radial organization of the neocortex in a gyrencephalic (C) and lissencephalic (D) species. (E) Phylogenetic tree schematizing distinct cortical folding patterns of different mammalian species. CB = cerebellum; CC = corpus callosum; CP = cortical plate; CTX = cortex; GM = gray matter; HIP = hippocampus; HYP = hypothalamus; LV = lateral ventricle; MB = midbrain; OB = olfactory bulb; STR = striatum; TH = thalamus; V-SVZ = ventricular-subventricular zone; WM = white matter.

The cerebral cortex, which is the central controller of higher-order cognitive functioning and sensory processing, includes three main brain regions: the neocortex, archicortex (hippocampus, dentate gyrus), and paleocortex. This review focuses on the neocortex, a mammalian-specific brain region that is evolutionarily the newest addition to the central nervous system. The mature neocortex comprises six radially organized neuronal layers (L1–L6) and, in the tangential plane, is subdivided into primary “areas” that are responsible for motor, somatosensory, auditory, and visual signal processing (Figure 1A). An additional parcellation into more than 50 anatomically and histologically distinct Brodmann areas fine-tunes neocortical functioning; for example, Brodmann area 17 encompasses the primary visual area of the cortex.

As the cerebral cortex has scaled in size in larger mammals, including in humans and nonhuman primates (NHPs), cortices acquired increasingly more complex gyrencephalic or highly folded forms, contrasting to the smooth, lissencephalic cortical surfaces observed in smaller mammals, including the oft-studied rodent models (Borrell and Reillo 2012) (Figure 1B). Indeed, all zones and layers are smooth in rodent cortices (Figure 1B). This absence of folding is considered an evolutionary adaptation referred to as “phyletic dwarfing” (Kelava and others 2013) since all mammals are thought to have arisen from a common ancestor with a gyrencephalic brain (Lewitus and others 2014; O’Leary and others 2013). In gyrencephalic species, folding is especially prominent within supragranular (i.e., upper) neuronal layers, whereas infragranular (i.e., lower) neuronal layers are relatively smoother and germinal zones are not folded (Garcia and others 2018) (Figure 1C). Gyrification involves the formation of both gyri (outward folds or ridges) and sulci (inward fissures). Evolutionary advantages of cortical folding include (1) the accommodation of an increased number of cortical neurons, especially supragranular neurons, providing an increased surface area (Fernandez and Borrell 2023), and (2) physical localization of functionally related neurons closer together to enable shorter axonal connections and more efficient wiring between cortical domains (Klyachko and Stevens 2003).

The great ape branch of the primate evolutionary tree is characterized by complex cortical folding patterns (DeCasien and others 2022). Within this branch, there exists further diversity; for example, humans have distinct cortical folding patterns compared to chimpanzees (Van Essen and others 2019) (Figure 1E). Marine mammals (cetaceans) also have complex patterns of cortical gyrification, resembling the intricacy observed in great apes, even though marine and terrestrial mammals diverged 52 million years ago (Butti and others 2011). Comparatively, folding is considerably less complex in Old World monkeys, including macaque, and lesser yet in New World monkeys, such as the marmoset (Figure 1E). Finally, cortical gyrification is also observed in some smaller mammals, such as carnivore ferrets, within which folding patterns are simplified and include mainly primary folds.

Understanding the molecular drivers of gyrification and differences across species is the subject of numerous studies, especially over the past few decades, leading to molecular and cellular insights into the drivers of cortical gyrification, which are reviewed in the succeeding sections. A tension-based, mechanical model of cortical folding has also gained support, which posits that axons, dendrites, and radial glial processes provide tension forces that influence cortical folding and overall tissue morphogenesis (Van Essen 2023; Van Essen and others 2019). These mechanical models, which have been extensively reviewed elsewhere (Bayly and others 2014; Kroenke and Bayly 2018; Tallinen and others 2014; Van Essen 2023; Van Essen and others 2019), provide important advances in our understanding of the biomechanics of cortical tissue morphogenesis. However, herein we focus on the molecular and cellular regulators of cortical gyrification.

### Understanding Cortical Development—A Roadmap from Rodent Models

**Figure 2.**
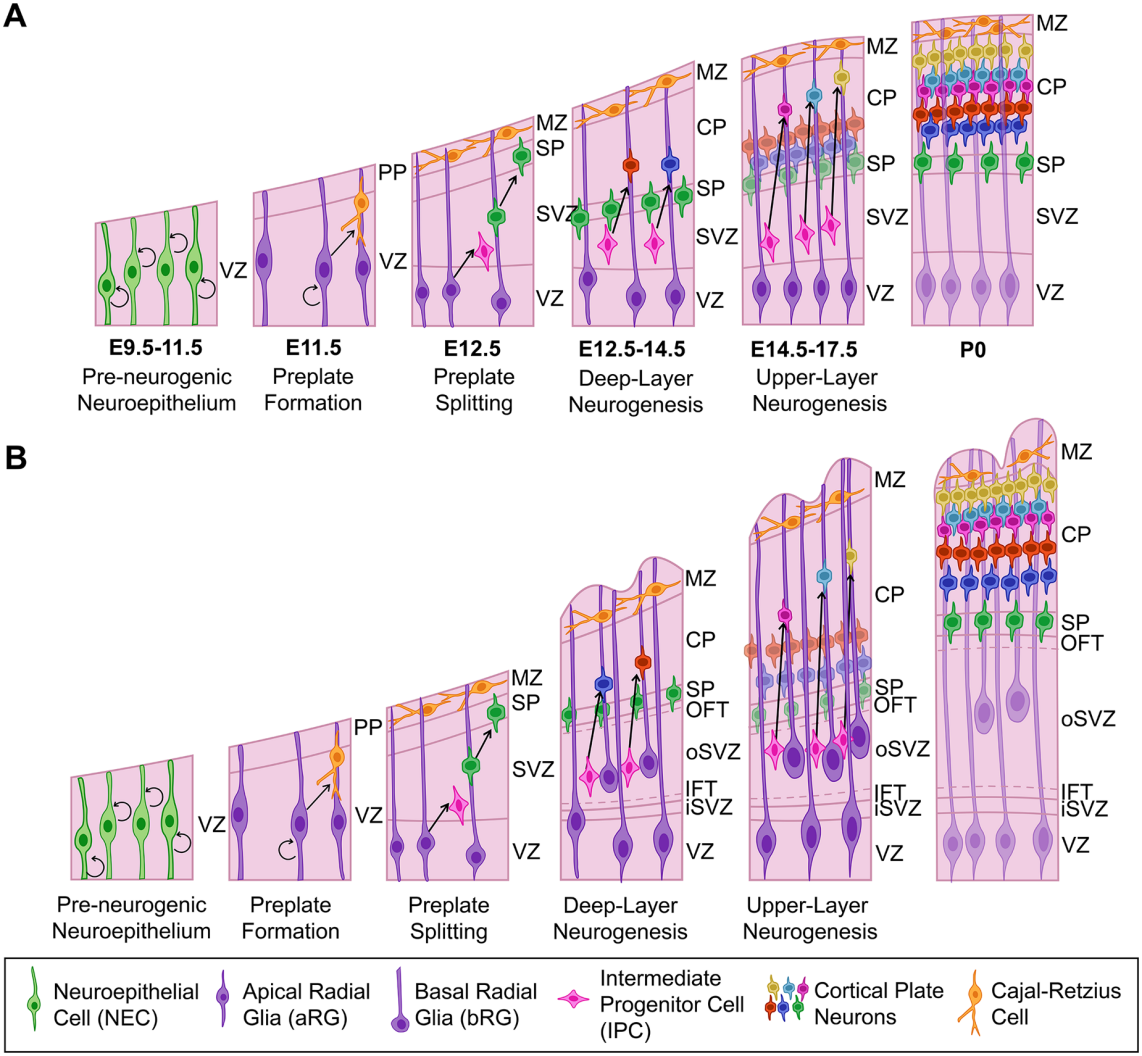
Development of murine and human/nonhuman primate (NHP) neocortex. (A) Murine neocortex begins as a neuroepithelium populated by NECs, which transform into neurogenic aRG at ~E10.5, when preplate neurons begin to differentiate. Deep-layer neurogenesis begins at ~E12.5, with layer 6 neurons splitting the preplate into an upper marginal zone and lower subplate. IPCs are also generated at this stage, leading to the formation of the SVZ. At ~E14.5, upper-layer neurons start to differentiate, with neurogenesis complete by E17. (B) Human and NHP primate corticogenesis is similar in the apical compartment, but the basal compartment has diversified. In particular, the SVZ is split into an iSVZ and oSVZ that are separated by an inner fiber tract. The oSVZ is populated by bRG and IPCs that have an enhanced proliferative and neurogenic potential, leading to the increased production of upper-layer neurons and cortical folding. aRG = apical radial glia; bRG = basal radial glia; CP = cortical plate; IFT = inner fiber tract; IPC = intermediate progenitor cell; iSVZ = inner subventricular zone; MZ = marginal zone; NEC = neuroepithelial cell; OFT = outer fiber tract; oSVZ = outer subventricular zone; PP = preplate; SP = subplate; SVZ = subventricular zone; VZ = ventricular zone.

Neocortical neurons are derived from dorsal telencephalic/pallial neural progenitor cells (NPCs), with layer 1 (marginal zone) and transient layer 7 (subplate) neurons born first, followed by the sequential, inside-out generation of cortical plate deep-layer (L) 6 and L5 neurons and then upper-layer L4–L2 neurons (Caviness and others 2009; Takahashi and others 1999) (Figure 2A). There are two cortical NPC compartments: apical and basal. Principal apical NPC types that reside in the cortical ventricular zone (VZ), include neuroepithelial cells (NECs) and ventricular or apical radial glia (aRG). Basal NPCs, which form a subventricular zone (SVZ), primarily include outer or basal radial glia (bRG) and intermediate progenitor cells (IPCs).

Prior to the onset of neurogenesis, the pallial neuroepithelium exclusively contains apical NECs, which form a neuroepithelial sheet that appears pseudostratified due to cell cycle–associated interkinetic nuclear migration: S-phase nuclei form an abventricular band on the basal side of the VZ, G2/M-phase nuclei divide at the apical surface, and G1-phase nuclei are in the central VZ. NECs divide symmetrically to expand the NPC pool (Taverna and others 2014) (Figure 2A). At E10.5, NECs transform into aRG, which differ from NECs in that they express several glial-specific genes (e.g., GLAST/Slc1a3, BLBP/Fabp7, GS/Glul) (Kriegstein and Götz 2003). Aside from differences in gene expression, aRG and NECs are similar, as both express the homeodomain transcription factor Pax6, display apicobasal polarity, extend bipolar processes that contact the adherens junction belt at the ventricular surface and the basal lamina at the pial side of the growing cortex, and undergo interkinetic nuclear migration (Taverna and others 2014). However, unlike NECs, aRG are neurogenic and switch to an asymmetric mode of division, giving rise to another aRG daughter cell and either a neuron or an IPC (Taverna and others 2014).

Basal NPCs lose their apical and, in some cases, basal contacts and migrate out of the VZ to form a SVZ (Borrell and Gotz 2014). IPCs, which are the predominant basal NPC type in rodents, lose ventricular and basal attachments (Haubensak and others 2004; Miyata and others 2004; Noctor and others 2004) (Figure 2A). IPCs also turn off Pax6 and initiate Eomes expression, a T-box transcription factor (aka Tbr2). In rodents, IPCs are the main contributors to neurogenesis, especially upper-layer neurons, and have a limited proliferative capacity, dividing once or twice before undergoing terminal, symmetric neurogenic divisions (Haubensak and others 2004; Miyata and others 2004; Noctor and others 2004). In contrast, bRG, which lose their apical process and retain their basal process, comprise merely 5% of the basal NPC pool (Martinez-Cerdeno and others 2012; Shitamukai and others 2011; Wang and others 2011). Notably, bRG in the murine dorsolateral cortex do not resemble bRG from gyrencephalic species at the transcriptomic level, and they are not highly proliferative (Fietz and others 2012; Florio and others 2015). However, a pool of Hopx-expressing bRG in the murine dorsomedial cortex are proliferative and retain a transcriptomic gene expression signature similar to human bRG (Vaid and others 2018). The existence of bRG in the murine dorsomedial cortex supports the idea that this region is evolutionarily more ancient and a remnant of the gyrencephalic brain of a common mammalian ancestor (Lewitus and others 2014; O’Leary and others 2013).

### Differences in Cortical Development in Gyrencephalic Species

Apical NPCs are less divergent than basal NPCs in gyrencephalic versus lissencephalic species. One notable difference in the apical NPC compartment, identified using human cerebral organoid technology, is that the transition from NEC to aRG takes longer and involves an intermediate transitory state (tNEC) in humans and NHPs (i.e., gorilla and chimpanzee), a transient state not observed in rodents (Benito-Kwiecinski and others 2021). Additionally, a pool of subapical NPCs that reside in the VZ but undergo mitoses away from the ventricular surface to produce bRG is more abundant in species with higher gyrification indices (Pilz and others 2013).

Elegant time-lapse imaging of slices from the embryonic macaque cortex reveals that the basal NPC pool is highly heterogeneous, with individual NPCs undergoing fate transitions between cell states (Betizeau and others 2013). However, for simplicity, we focus on the general categories of bRG and IPCs, which are increased in number in gyrencephalic versus lissencephalic cortices (Fietz and others 2010; Reillo and others 2011) (Figure 2B). To accommodate a larger basal NPC pool, the SVZ housing these cells is subdivided into a smaller inner (iSVZ) and larger outer (oSVZ) compartment, separated by an inner fiber tract (Borrell 2018). The oSVZ is formed during a brief expansive phase when bRG are produced from aRG in symmetric, self-depleting divisions (Martinez-Martinez and others 2016). Studies in gyrencephalic species, including macaque (Betizeau and others 2013), ferret (Martinez-Martinez and others 2016), and humans (Hansen and others 2010; LaMonica and others 2013), have demonstrated that bRG can also self-amplify, dividing at least a couple of times. Since bRG also preferentially generate upper-layer neurons, unsurprisingly, the resulting gyri are larger and more exaggerated in upper layers of the cortex (Llinares-Benadero and Borrell 2019).

IPCs in gyrencephalic cortices have been likened to transit amplifying cells, a hallmark of which is to divide multiple times before differentiating, thus allowing IPCs to become major contributors to upper-layer neuronal expansion (Fietz and others 2010). Evidence supporting the critical role for IPCs in gyral formation comes from the deletion of Eomes, a signatory marker of IPCs, the loss of which suppresses cortical folding in the ferret cortex (Toda and others 2016). Additional support for the unique role of human IPCs in cortical folding comes from the analysis of histone H3 lysine 9 acetylation (H3K9ac), which is elevated in human versus murine basal NPCs (Kerimoglu and others 2021). Strikingly, when histone H3 acetylation is artificially increased in the murine cortex, IPCs expand and cortical folding ensues (Kerimoglu and others 2021), suggesting that differences in gene expression lie at the heart of the capacity for IPC self-renewal.

The initial hypothesis was that basal NPCs drive cortical gyrification through the expansion of upper-layer neuron production (Kriegstein and others 2006). However, in the rhesus monkey, cortical neurogenesis begins between E38 and E40 and continues until E70 or up to E102 in the limbic and visual cortices, respectively (Rakic 1974, 2002). In contrast, rapid surface area expansion and prominent cortical folding does not ensue until E102 (Wang and others 2017). Thus, cortical folding largely occurs after the neurogenic period in the rhesus monkey, suggesting that neurogenesis is not the main driver of cortical folding. An alternative hypothesis for bRG driving cortical gyrification was proposed based on the observation that the expanded oSVZ gives rise to more glial cells in sites where cortical gyri will form (Shinmyo and others 2022). Indeed, astrocytes are increased in number in naturally gyrencephalic species (Herculano-Houzel 2014). Moreover, in the macaque cortex, folding is initiated after bRG in the oSVZ switch from generating upper-layer neurons to producing astrocytes and oligodendrocytes (Rash and others 2019). Finally, blocking astrogenesis in the ferret cortex by inhibiting retinoic acid signaling, or eliminating cortical astrocytes by driving diphtheria toxin A (DTA) expression, reduces sulcal depth and gyral size (Shinmyo and others 2022).

### Radial Glial Scaffold and Neuronal Migration

In addition to serving as NPCs, the basal processes of apical and basal RG serve as structural guides along which newborn neurons migrate. In lissencephalic species, the RG scaffold is primarily composed of basal processes from aRG that attach to the basement membrane at the pial surface of the brain to facilitate glial-guided neuronal locomotion. Conversely, radial trajectories in gyrencephalic species are more irregular, with radial glia curving in the vicinity of developing sulci (Llinares-Benadero and Borrell 2019). Indeed, the radial fiber scaffold transforms into a fanned array in gyrencephalic cortices due to irregular interspersion of bRG and aRG processes (Reillo and others 2011), with more bRG processes contributing to the radial glial meshwork where gyri (ridges) form, and less bRG fibers observed in presumptive sulci (fissures) (Borrell 2018). Moreover, radially migrating excitatory neurons in ferret cortices have extensively branched leading processes that allow them to switch between radial fibers, further complexifying neuronal migratory patterns (Martínez-Martínez and others 2019).

One of the first pieces of evidence that neuronal migration might be a driving force for cortical folding came from revelations that mutations in *RELN*, encoding a glycoprotein secreted by Cajal-Retzius neurons in L1, are associated with a lissencephalic phenotype in humans (Amin and Borrell 2020; Hong and others 2000). In *Reeler* mutant mice that carry a spontaneous mutation in *Reln*, an inverted cortex forms, such that neurons with an “upper-layer” identity are at the bottom of the cortex and “lower-layer” neurons are at the top (Lambert de Rouvroit and Goffinet 1998). A striking disruption of inside-out neuronal migration is also associated with lissencephaly in humans (Chang and others 2007), most likely because *RELN* is also required to maintain the radial glial scaffold (Hartfuss and others 2003). Several other mutations have been discovered that alter the folding pattern of human cortices, resulting in severe neurologic disorders, as discussed further in Box 1.

**Box 1.** Insights into Cortical Folding from Human Disease Models.

**Figure 3.**
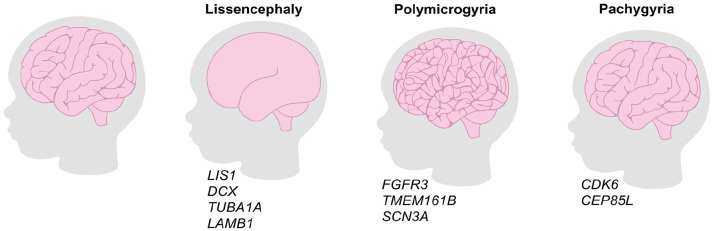
Genetic mutations associated with alterations to cortical folding in human disorders. Lissencephaly refers to a loss of cortical folding and is associated with mutations in *LIS1*, *DCX*, *TUBA1A*, and *LAMB1.* Polymicrogyria refers to an increase in cortical folding and is associated with mutations in *FGFR3*, *TMEM161B*, and *SCN3A.* Pachygyria is associated with a reduction but not loss in cortical folding and is associated with mutations in *CDK6* and *CEP85L.*

Defects in cortical folding are associated with severe neurologic disorders, including epilepsy, schizophrenia, autism, and intellectual disabilities (Fernández and others 2016) (Figure 3). Different types of folding disorders have been documented, including a loss of folds (lissencephaly), reduction in folds (pachygyria), and an increase in folds (polymicrogyria), each associated with distinct genetic mutations. (1) Lissencephaly: Point mutations in *RELN* are associated with lissencephaly, emphasizing its critical role in guiding the migration of cortical neurons during development (Chang and others 2007). Miller-Dieker syndrome (MDS) is another classical lissencephaly disorder that is associated with mutations in *LIS1* (Dobyns and others 1999). *LIS1* interacts with the centrosome to control the migration of cortical neurons; mutations in this gene are associated with a disruption of microtubules in migrating neurons (Feng and others 2000). Other genes mutated in human patients with classical lissencephalies also encode microtubule-related proteins, including *DCX* and *TUBA1A* (Liu and others 2012). Cobblestone lissencephaly is a cortical migration disorder associated with the mutation of genes that make up the basal lamina, including *LAMB1*, encoding for laminin beta-1 and laminin γl114, and perlecan. These disorders are associated with a disruption of the basement membrane, allowing neurons to migrate outside what is usually a limiting membrane to form bulges on the brain surface (Amin and Borrell 2020). (2) Pachygyria: The loss of expression or misexpression of mutant versions of several genes that regulate neuronal migration results in a reduction in folding instead of a complete loss of cortical folds. In humans, mutations in *CEP85L* cause pachygyria, and similar to *LIS1*, this phenotype is due to a disruption of the centrosome and microtubule cytoskeleton (Kodani and others 2020). A similar reduction in folds is seen in microcephaly primary hereditary (MCPH), or primary microcephaly, and is associated with mutations in *CDK6*. Patients with MCPH have smaller brains with a reduced complexity of cortical folds (Wang and others 2022). Experimentally, the MCPH-associated mutation in *CDK6* prevents *CDK6* bRG expansion (Wang and others 2022). (3) Polymicrogyria: Patients with thanatophoric dysplasia, caused by a constitutive gain-of-function mutation in *FGFR3*, have increased cortical folding, or polymicrogyria. Notably, hyperactive Fgf signaling in ferret recapitulates the increase in gyral formation and is attributed to an increase in bRG and IPCs (Masuda and others 2015). Similarly, biallelic mutations in *TMEM161B* are associated with polymicrogyria, which disrupts primary cilia and Shh signaling, leading to intellectual disability and seizures (Akula and others 2023). Interestingly, a mutation in *SCN3A* that encodes for a voltage-gated sodium channel also results in polymicrogyria, providing the first evidence that a channelopathy can affect brain structure and neuronal migration (Smith and others 2018).

## Intrinsic Cues That Regulate Cortical Folding

### Focal Increases in Proliferation Induce Cortical Folding in the Lissencephalic Rodent Brain

**Figure 4.**
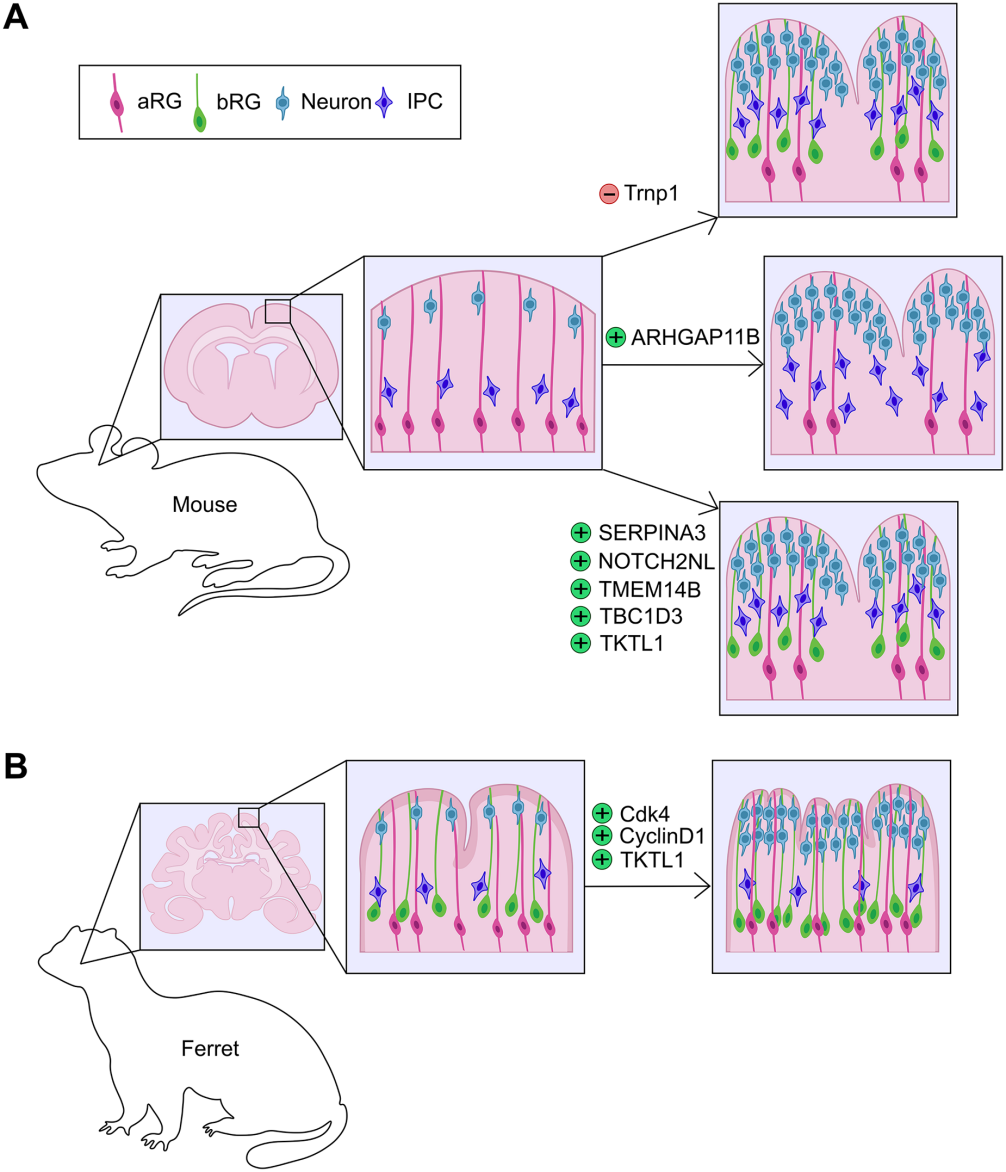
Genetic manipulations in rodent and ferret cortices that affect folding. (A) Genetic manipulations in rodent cortices that lead to the formation of gyral-like structures via the expansion of bRG include the focal knockdown of *Trnp1* and the overexpression of human or nonhuman primate–specific genes, including *SERPINA3*, *NOTCH2NL*, *TMEM14B*, *TBC1D3*, and *TKTL1. ARHGAP11B* overexpression also leads to cortical folding in rodents but through the overproduction of IPCs. (B) Genetic manipulations that increase cortical folding in ferret include the overexpression of *Cdk4*, *CyclinD1* or *TKTL1*. aRG = apical radial glia; bRG = basal radial glia; IPC = intermediate progenitor cell.

Local proliferative hot spots in the cortical germinal zones are observed in several gyrencephalic animals, including ferrets, cats, and humans, and are thought to prefigure gyral formation (Reillo and others 2011). Indeed, there is growing support for the idea that asymmetric patterns of proliferation induce cortical gyration. First, bRG are more prevalent in sites where cortical gyri will form, especially bRG that express HOPX (Matsumoto and others 2020). HOPX is a homeodomain-only transcription factor commonly expressed in human, monkey, and ferret bRG (Johnson and others 2018; Pollen and others 2015). While HOPX-negative bRG also exist, they tend to exit the cell cycle and divide less frequently than HOPX^+^ bRG (Matsumoto and others 2020). Second, genetic manipulations that increase NPC proliferation in focal sites in the murine cortex induce the formation of fold-like structures. For example, local knockdown of *TMF-regulated nuclear protein* (*Trnp1*) expands subapical progenitors and basal NPCs, leading to cortical folding in mouse (Pilz and others 2013; Stahl and others 2013) (Figure 4A) and enhanced gyral formation in ferret (Martinez-Martinez and others 2016) (Figure 4B). Overexpression of proliferation-promoting genes, such as the cell cycle regulators *Cdk4* and *Cyclin D1*, promotes bRG proliferation and surface area expansion in the context of a naturally gyrencephalic cortex, leading to overfolding of the ferret brain (Nonaka-Kinoshita and others 2013) (Figure 4B). In contrast, the uniform overexpression of *Cdk4* and *Cyclin D1* does not induce folding in the murine cortex (Nonaka-Kinoshita and others 2013). In addition, untethering of the centrosome of aRG from the ventricular surface by mutating the centrosomal gene Cep83 leads to overproliferation of aRG, increasing the production of IPCs, deep- and upper-layer neurons, and the appearance of cortical folds in a rodent model (Shao and others 2020).

New insights into the molecular drivers of cortical folding originate from the identification of cortically expressed genes that are human or NHP specific (Florio and others 2018). Throughout evolution, genes have evolved to drive new developmental pathways via various mechanisms, including de novo origins, gene duplications to create new gene paralogs, or alterations to existing gene sequences that change the structure/function of ancestral gene products or modify their regulatory regions and alter gene expression patterns (Bitar and others 2019). Examples of newly evolved genes that influence cortical gyrification include (1) *TMEM14B*, a primate-specific gene paralog, the focal overexpression of which increases bRG and IPC numbers and upper-layer neurogenesis, leading to gyral formation in the rodent brain (Liu and others 2017) (Figure 4A). Notably, *TMEM14B*-driven expansion of bRG is correlated with a shift to more oblique aRG divisions, which naturally, preferentially occur in aRG that give rise to bRG (LaMonica and others 2013; Shitamukai and others 2011). (2) Local overexpression of *TBC1D3*, a hominoid-specific gene, in the murine cortex induces aRG to delaminate and form an expanded bRG pool, triggering gyral formation (Ju and others 2016) (Figure 4A). Conversely, *TBC1D3* down-regulation in human cortical slices reduces bRG number (Ju and others 2016). (3) *ARHGAP11B*, a human-specific gene that induces aRG to divide symmetrically to produce two IPCs, expands the IPC pool and promotes folding of the murine cortex (Florio and others 2015) (Figure 4A). *ARHGAP11B* overexpression can also increase cortical folding in marmosets and ferrets by increasing bRG numbers and upper-layer neurogenesis (Heide and others 2020; Kalebic and others 2018). (4) Human-specific *NOTCH2NL* paralogs drive basal NPC proliferation and cortical folding, as described in more detail in the extrinsic signaling section below (Fiddes and others 2018; Florio and others 2018; Suzuki and others 2018) (Figure 4A).

Along with genes that have newly evolved to support cortical folding, other genes have incurred minor changes to their existing sequence or upstream regulatory regions: (1) *SERPINA3* is a conserved gene that has acquired a new site of expression in human bRG during cortical evolution (Zhao and others 2022). In contrast, murine *Serpina3n* is not expressed in the developing murine neocortex (Zhao and others 2022). Overexpression of human *SERPINA3* in the murine cortex increases bRG number and enhances upper-layer neurogenesis, leading to focal gyration (Zhao and others 2022) (Figure 4A). Strikingly, *SERPINA3-*overexpressing mice survive into adulthood and demonstrate enhanced learning and memory in behavioral tests (Zhao and others 2022). (2) An intronic *cis*-regulatory element (CRE) in the TRNP1 locus has undergone mutations to produce de novo binding sites for several transcription factors, including CTCF; alterations are observed in Old World monkeys, apes, and humans (Kliesmete and others 2023). Moreover, increased Trnp1 expression is initiated when histone H3 acetylation is elevated, driving IPC expansion and cortical folding in the murine cortex (Kerimoglu and others 2021). (3) *TKTL1*, encoding a transketolase, carries a single amino acid substitution in modern-day humans that is absent in NHP or extinct humans (i.e., Neanderthals and Denisovans) (Pinson and others 2022). When overexpressed in the murine or ferret brain, the human-specific *TKTL1* allele increases bRG proliferation and upper-layer neurogenesis (Figure 4A), while conversely, deletion of *TKTL1* from human fetal cortices or cerebral organoids using CRISPR gene-editing reduces bRG cell division (Pinson and others 2022). Taken together, these findings not only cement the link between bRG expansion and cortical folding but also provide evidence that gyrification has evolved due to genetic changes that are correlated with enhanced cognitive abilities in modern-day humans.

### Regional Heterogeneity in Gene Expression across the Germinal Zone: A Driver of Neurogenic Asynchrony

**Figure 5.**
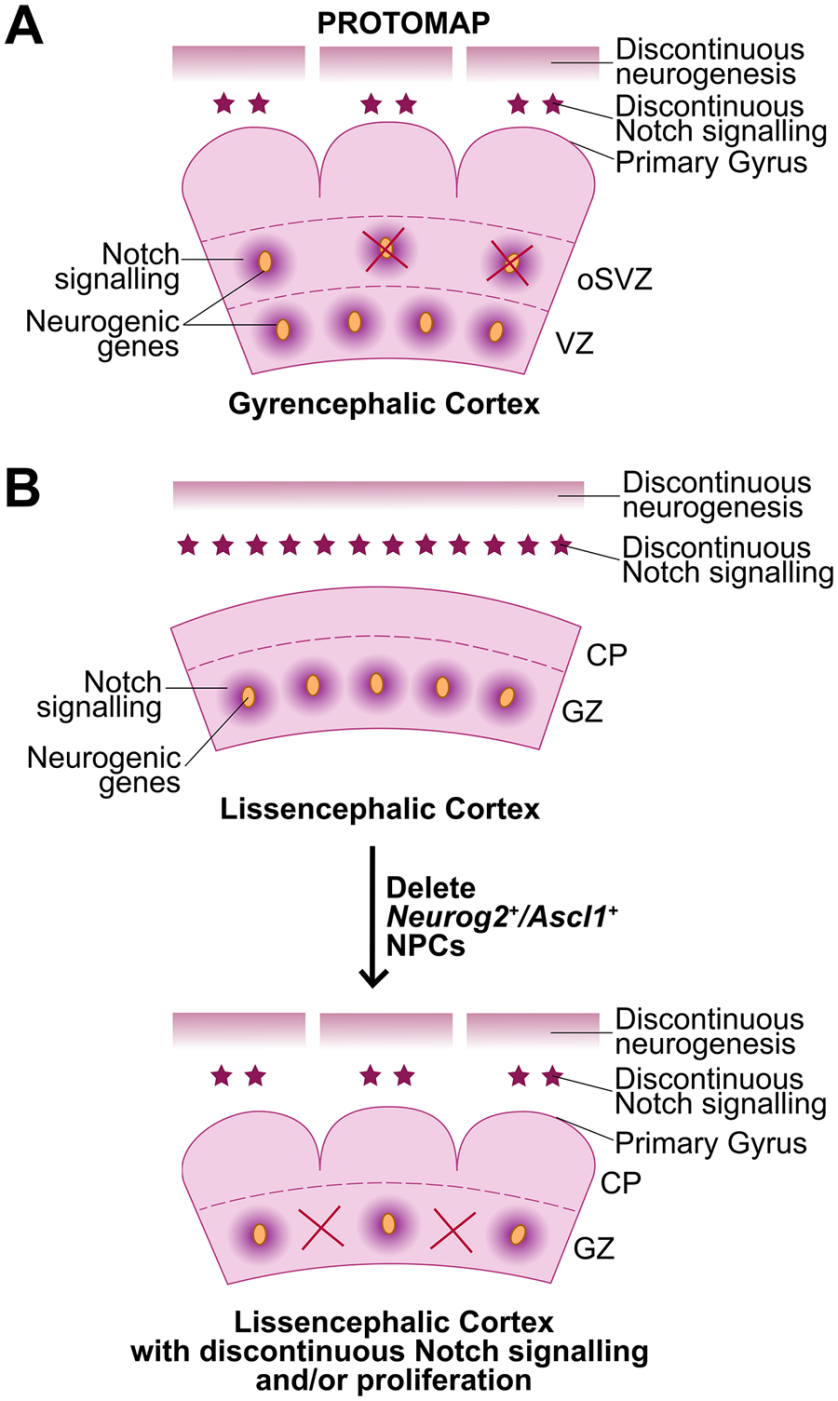
Protomap model of cortical folding. (A) A transcriptomic protomap of neurogenic genes prefigures discontinuous patterns of Notch signaling and neurogenesis, especially in the oSVZ, in gyrencephalic ferrets. (B) The protomap is not present in the lissencephalic rodent cortex, where neurogenic genes and Notch signaling molecules are uniformly expressed. (C) The deletion of *Neurog2*^+^*/Ascl1*^+^ “niche” cells, which express high levels of Notch ligands, leads to a discontinuous pattern of Notch signaling and neurogenesis, leading to cortical folding and mimicking the protomap observed in gyrencephalic cortices. CP = cortical plate; GZ = germinal zone; oSVZ = outer subventricular zone; VZ = ventricular zone.

Primary gyri and sulci form in stereotyped locations, followed by the further elaboration of secondary and tertiary folds (Sun and Hevner 2014). In human cortices, the formation of primary folds begins at week 27 of gestation, followed by secondary folding at 31 weeks and final tertiary folding at 37 weeks (Garcia and others 2018). An underlying network of molecular events must thus precisely determine where “hot spots” of bRG proliferation and upper-layer neurogenesis are located (Reillo and others 2011). In a breakthrough study, transcriptomic data of ferret germinal zones corresponding to the presumptive splenial gyrus and abutting lateral sulcus revealed a genetic protomap that prefigures cortical folding (de Juan Romero and others 2015). Differentially expressed genes (DEGs) between presumptive gyri and sulci are most abundant in the oSVZ and are enriched in gene ontology categories associated with the cell cycle, neurogenesis, and fate specification (de Juan Romero and others 2015). Of these DEGs, neurogenic genes are expressed at elevated levels in the oSVZ where presumptive gyri will form, while cell cycle–associated genes are expressed at higher levels within presumptive sulci (de Juan Romero and others 2015) (Figure 5A). Intriguingly, 80% of these DEGs are associated with human cortical malformations (de Juan Romero and others 2015), suggesting that many important drivers of neurogenic patterning and gyral formation may be mined from this data set. In contrast, an equivalent protomap does not exist in the murine cortex, which does not undergo cortical folding (de Juan Romero and others 2015).

Recently, the proneural genes *NEUROG2* and *ASCL1*, and the neurogenic gene *HES1*, a readout of active Notch signaling, were shown to have a modular transcript distribution in the cortical germinal zone in macaque (Han and others 2021) (Figure 5A). This modular pattern contrasts with the uniform expression of other aRG/bRG markers, such as PAX6. Since *NEUROG2* and *ASCL1* are essential drivers of cortical neurogenesis, while conversely, Notch signaling genes maintain the proliferative status of cortical NPCs (Han and others 2021; Oproescu and others 2021), their modular patterns of expression were predicted to contribute to the asymmetric patterns of neurogenesis observed in gyrencephalic species. Consistent with this prediction, the specific deletion of *Neurog2/Ascl1* double^+^ NPCs, which serve as Notch-ligand expressing niche cells, induces cortical folding in mice (Han and others 2021). Notably, the underlying cause of folding is not basal NPC expansion but rather a change in the pattern of neurogenesis, which becomes asynchronous across the cortical wall. This study supports the notion that cortical folding is prefigured by sites of high and low neurogenesis (Han and others 2021).

## Extracellular Signaling Molecules Induce Cortical Folding

### Signaling Molecules Act Non–Cell Autonomously to Affect Cortical Folding

#### Notch signaling

**Figure 6.**
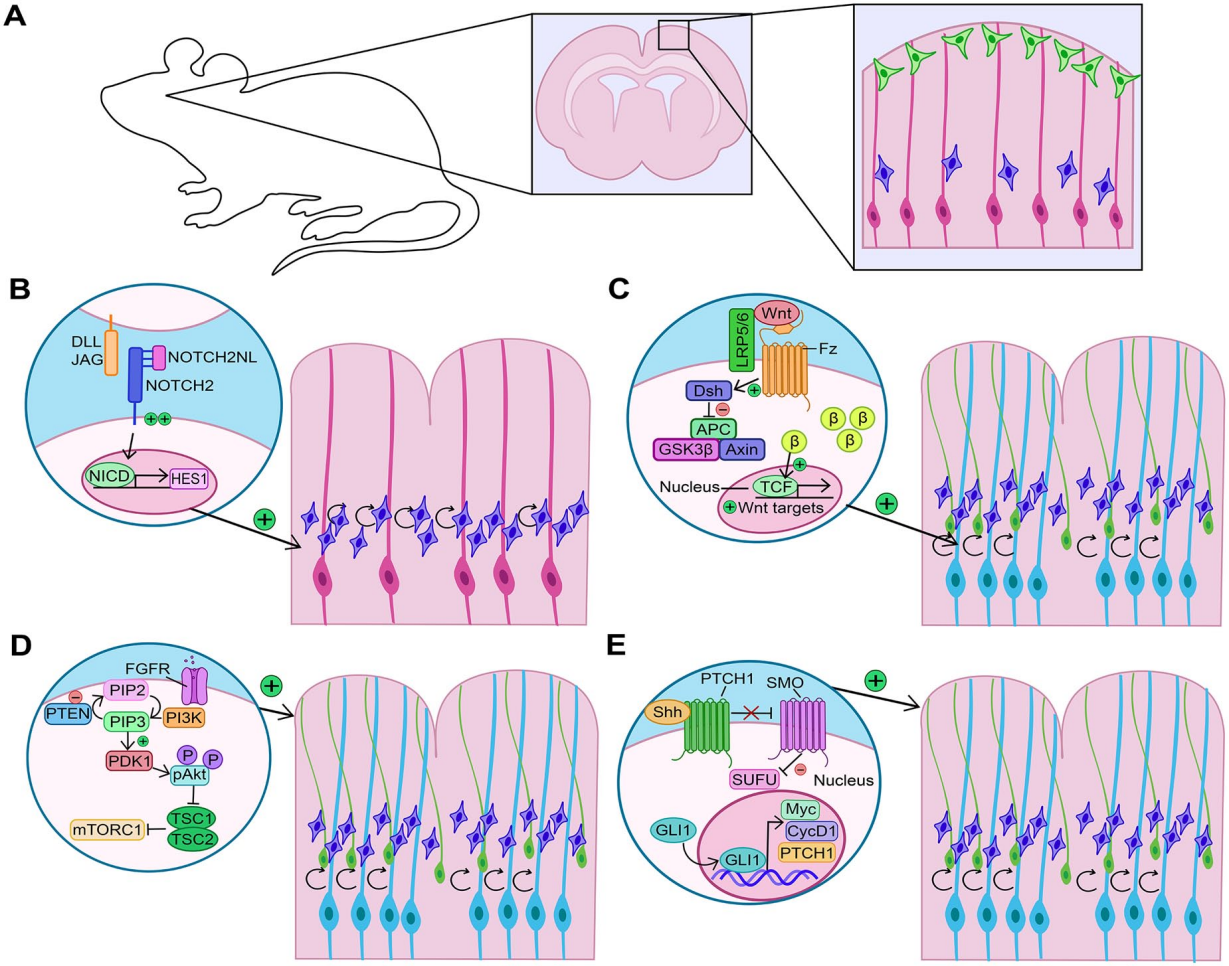
Alterations to signaling pathways that affect cortical folding. (A) Rodent cortex comprises two principal neural progenitor cell pools: apical radial glia (aRG) (pink cells) and intermediate progenitor cells (IPCs) (blue cells), which differentiate into cortical neurons (green cells). (B) Increased Notch signaling expands the IPC pool, leading to cortical folding. (C) Increased Wnt signaling first expands the aRG pool (light blue), which then give rise to more IPCs and basal radial glia (bRG) to increase cortical neurogenesis and induce folding. (D) Increased FGF signaling promotes an expansion of aRG, bRG, and IPCs to increase neurogenesis as well as astrocytogenesis, leading to cortical folding. PI3K is a downstream pathway similarly implicated in driving cortical folds when focally overactivated. (E) Shh signaling can induce cortical folding by driving an expansion of aRG, bRG, and IPCs to increase neurogenesis.

The first evidence that Notch signaling could be vital for cortical folding in gyrencephalic species were correlative studies demonstrating that Notch signaling genes are differentially expressed in developing sulci and gyri of the ferret neocortex (de Juan Romero and others 2015; Han and others 2021). These studies suggested that Notch might provide a niche-like signal to control NPC proliferation and expansion (de Juan Romero and others 2015; Han and others 2021). Candidate genes include four human-specific paralogs of the *NOTCH2* receptor (*NOTCH2NL-A,-B,-C,-R*) found on chromosome 1q21, a locus that underwent a large-scale pericentric inversion during human evolution, with associated gene loss and duplication events (Fiddes and others 2018). Notably, 1q21 deletion and duplication syndromes are associated with microcephaly and macrocephaly, respectively, and cortical-specific expression of the aforementioned *NOTCH2* paralogs makes these genes prime candidates as etiological factors (Fiddes and others 2018). While NOTCH2NL paralogs retain extracellular domains that bind Delta ligands, they lack transmembrane and cytoplasmic regions and are secreted (Fiddes and others 2018). As a secreted protein, NOTCH2NL competitively binds DLL1 to reduce Delta ligand accessibility at the cell membrane (Suzuki and others 2018). Nevertheless, the truncated NOTCH2NL receptor can heterodimerize with full-length NOTCH2 receptors to enhance Notch signaling, such that *NOTCH2NL* overexpression induces Notch effector gene expression (e.g., *HES1*) (Fiddes and others 2018; Suzuki and others 2018) (Figure 6A,B).

*NOTCH2NL* paralogs are expressed in human fetal cortical cells, including at elevated levels in bRG (Fiddes and others 2018). When overexpressed in vitro, *NOTCH2NL* promotes the expansion of cortical NPCs generated from human embryonic stem cells (hESCs) in two-dimensional culture and inhibits neurogenesis in three-dimensional hESC-derived cerebral organoids (Suzuki and others 2018). When overexpressed in the embryonic mouse cortex, *NOTCH2NL* increases cycling of basal NPCs, most notably, Eomes^+^ IPCs (Fiddes and others 2018; Florio and others 2018) (Figure 6A,B). On the other hand, Notch signaling is required to support the proliferation of bRG, demonstrated by pharmacologic inhibition in human cortical explants that induces bRG to stop proliferating and differentiate (Hansen and others 2010). Furthermore, the generation of cerebral organoids from hESCs containing homozygous CRISPR/Cas9-knockouts of *NOTCH2NLA* and *NOTCH2NLB*, alongside a heterozygous loss of *NOTCH2NLC*, revealed a reduction in size and increase in neuronal differentiation genes (Fiddes and others 2018). While these studies do not directly support a role for *NOTCH2NL* genes in cortical folding, their ability to affect basal NPC proliferation/differentiation decisions makes it highly likely.

#### Wnt signaling

Wnt activation induces aRG to undergo symmetric, self-renewing divisions, delaying the generation of basal NPCs (Chenn and Walsh 2002; Draganova and others 2015; Munji and others 2011). Cortices in which Wnt signaling is hyperactive appear gyrated, but they are not true folds, as they primarily form in the germinal zone and arise due to expansion of the aRG pool (Chenn and Walsh 2002). Consistent with these findings, miR-3607, which increases Wnt signaling by degrading APC transcripts, part of the β-catenin destruction complex, is expressed in aRG in gyrencephlic species and not in rodents (Chinnappa and others 2022). Overexpression of miR-3607 in the rodent cortex or human cerebral organoids induces aRG proliferation and a similar expansion of the VZ, while conversely, loss of miR-3607 hampers aRG expansion in ferret (Chinnappa and others 2022). In comparison, deletion of *Ctnnb1*, a Wnt effector gene, leads to bRG and iPC expansion in mice, but only in a transient early window, likely due to premature depletion of the aRG pool (Draganova and others 2015). The “sweet spot” of Wnt signaling occurs in *Lmx1a;Lmx1b* double knockout mice, in which a transient increase in Wnt signaling at early stages of cortical development expands the aRG pool, followed by a decline in Wnt signaling that allows these aRG to give rise to Eomes^+^ IPCs and Pax6^+^ bRG (Chizhikov and others 2019) (Figure 6C). Strikingly, in these *Lmx1a;Lmx1b* double knockouts, neuronal layer folding closely resembles that of gyrencephalic cortices (Chizhikov and others 2019). Interestingly, *Lmx1a/Lmx1b* are not expressed in cortical NPCs themselves; rather, they control long-range signaling that affects cortical NPCs through their regulation of BMP signaling and requirement to pattern the cortical midline (Chizhikov and others 2019).

#### Fibroblast growth factor signaling

Fibroblast growth factor (Fgf) signaling is required to support cortical NPC proliferation in rodents (Rash and others 2011). Interestingly, a constitutively active mutant allele of *FGFR3* (K644E) that is associated with polymicrogyria in humans promotes cortical expansion without folding when expressed in mice (Inglis-Broadgate and others 2005; Lin and others 2003). In contrast, intraventricular injection of FGF2 induces cortical folding in mouse, potentially through the induction of focal hot spots of proliferation (Rash and others 2013). Similarly, when Fgf8 is expressed in the naturally gyrencephalic ferret cortex, the basal NPC pool is expanded and cortical folding is enhanced (Masuda and others 2015) (Figure 6D). Conversely, when FGF signaling is inhibited in the naturally gyrencephalic ferret brain, there is a reduction in Pax6^+^ bRG, reduced upper-layer neurogenesis, and impaired cortical folding (Matsumoto and others 2017).

In addition to regulating NPC proliferation, FGF signaling has been implicated in the expansion of cortical astrocytes (Shinmyo and others 2022). Focal increases of FGF signaling in the murine cortex induce astrocyte proliferation and expansion without affecting neuronal number, leading to gyrus-like formations (Shinmyo and others 2022) (Figure 6D). Astrocytes appear to be the drivers of cortical folding since they are naturally more numerous in gyri, and their elimination in the ferret cortex lessens overall folding by decreasing vertical expansion (Shinmyo and others 2022).

#### Phosphoinositide 3-kinase signaling

Phosphoinositide 3-kinase (PI3K) is a central signal transduction molecule that converts phosphatidylinositol (3,4)-bisphosphate (PIP2) to phosphatidylinositol (3,4,5)-trisphosphate (PIP3), a membrane phospholipid that serves as a second messenger and activates downstream signaling. PI3K and PIP3 control multiple cellular processes, including cell polarity, proliferation, and migration (Comer and Parent 2007). Cortical dysplasia-resembling folds are observed in transgenic mouse models overexpressing constitutively active alleles of Pik3ca, the catalytic subunit of PI3K, which are associated with overgrowth disorders in humans (i.e., *PIK3CA*-*H1047R* and *E545K*) (Roy and others 2015). Additionally, transient overexpression of the *H1047R* allele in a two-day window between E13.5 and E15.5 induces gyrencephaly in mice (Roy and others 2019) (Figure 6D). The formation of cortical folds is presaged by a loss of apical adhesion and elevated Yap activity, which is a central driver of the folding phenotype (Roy and others 2019). Further supporting the role of PI3K in cortical folding is the finding that PTEN mutant human cerebral organoids, with elevated PI3K signaling, have increased surface folding (Li and others 2017).

#### Sonic hedgehog signaling

Shh is a morphogen that establishes dorsoventral patterning in the embryonic CNS and, later, plays an important role in the establishment of cortical gyration. Elevating Sonic hedgehog (Shh) signaling in the lissencephalic mouse cortex, using a constitutively active Smoothened (SmoM2) allele, leads to increased upper-layer neuron production and folding of the cingulate cortex (Wang and others 2016). Interestingly, increases in upper-layer neuron production are associated with an increased proliferative capacity of IPCs and bRG, whereas aRG preferentially form bRG at the expense of IPCs (Wang and others 2016) (Figure 6E). Notably, IPCs produced in response to high Shh signaling have a higher proliferative capacity (Wang and others 2016). The impact of Shh on bRG expansion was further recapitulated in human cerebral organoids upon application of a SMO agonist, inducing bRG expansion and increasing the number of SATB2 upper-layer neurons (Wang and others 2016).

The role of Shh signaling in cortical folding has also been investigated in the gyrencephalic ferret brain (Matsumoto and others 2020). In ferrets, Shh signaling is most active in HOPX^+^ bRG; Shh is both necessary and sufficient to support bRG proliferation and upper-layer neurogenesis, thereby influencing subsequent folding (Matsumoto and others 2020). The targeted disruption of Shh in the embryonic ferret brain reduces bRG proliferation and inhibits gyral formation (Matsumoto and others 2020). Notably, SHH depends on CDK6 to expand the bRG pool in mice, ferrets, and human cerebral organoids, an activity that is independent of the kinase activity of CDK6 (Wang and others 2022).

Within neural cells, Shh signaling is compartmentalized to primary cilia, which are 1- to 10-µm cellular protrusions that are rich in growth factor and morphogen receptors and serve to sense signaling molecules (Nishimura and others 2019). Shh signaling requires cilia, since mutation of Kif3a, which is required for cilia formation, blocks basal NPC expansion and cortical folding (Wang and others 2016). Additional mutations that disrupt primary cilia similarly abrogate Shh signaling and perturb cortical gyration. *TMEM161B* expression is enriched in embryonic NPCs, including HOPX^+^ oRG. In *Tmem161b* null mice, primary cilia are disrupted, resulting in defective Shh signaling along with abnormal brain and spinal cord patterning (Akula and others 2023). Furthermore, *TMEM161B* knockdown in the developing gyrencephalic ferret brain disrupts the formation of cortical gyri and sulci. *Tmem161b* knockdown similarly affects neurogenesis and neuronal migration in a lissencephalic rodent model, biasing murine cortical NPCs at E14.5 toward lower- rather than upper-layer fates (Akula and others 2023). *TMEM161B* thus provides a critical link between Shh signaling and cortical gyrification.

## Mechanical Cues That Drive Cortical Folding

### Extracellular Matrix and Cortical Folding

Most tissues display structural anisotropy, meaning that component cells and the surrounding extracellular matrix (ECM) are precisely organized in three dimensions to support tissue function. The asymmetrical organization of the brain in three dimensions allows for a parcellation of tasks to tissue substructures. But how does tissue anisotropy and asymmetry arise in the developing cortex? The ECM is an important tissue component, as it forms a meshwork of proteins and proteoglycans that provide structural support and modulate cell signaling (Box 2). The ECM can thus directly control the mechanical properties of a cell, alter cell fate decisions or cell state transitions, and/or direct tissue self-assembly.

**Box 2.** An Introduction to the Extracellular Matrix (ECM).

**Figure 7.**
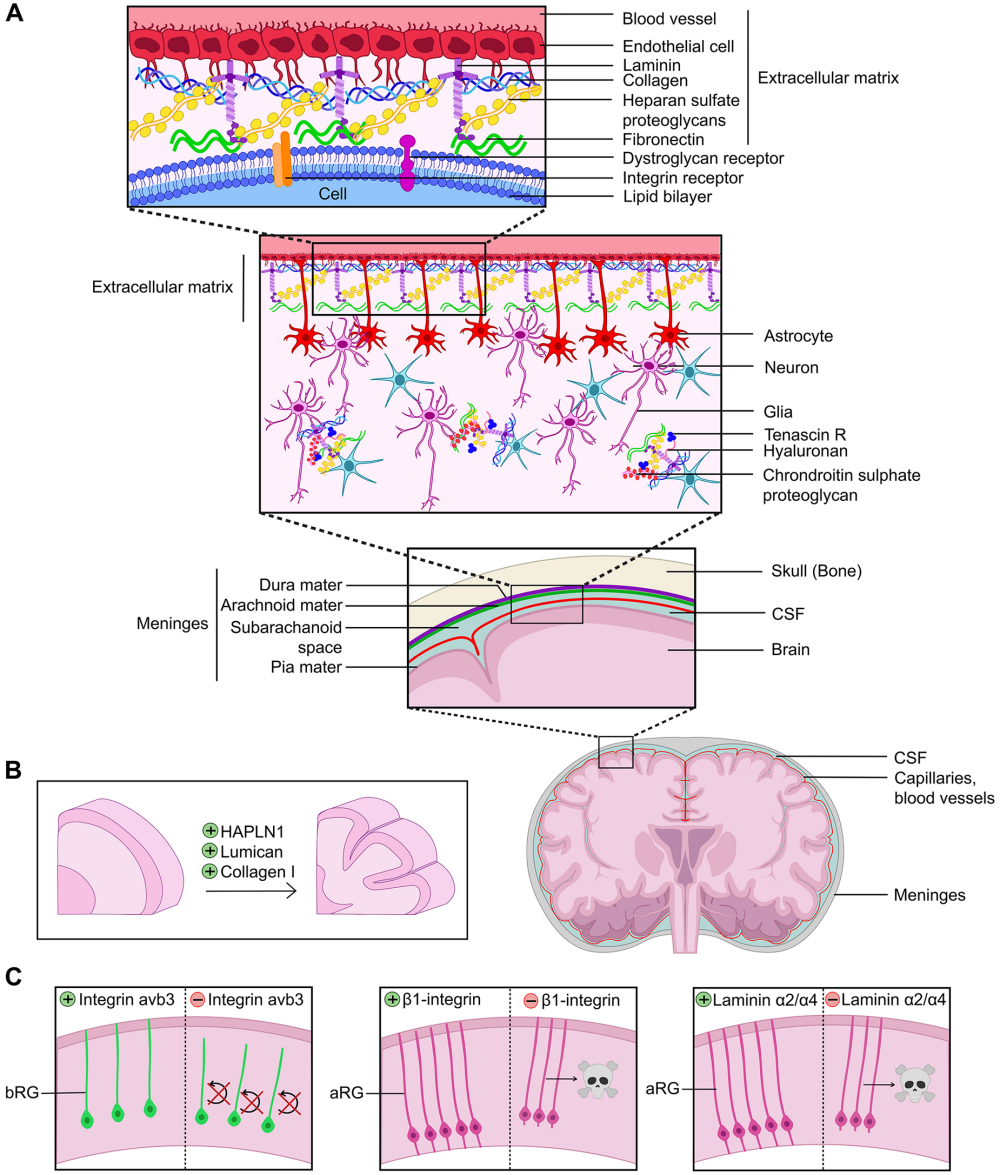
Role of the extracellular matrix (ECM) in cortical folding. (A) Structure of the ECM at the pial surface of the brain. (B) ECM molecules applied to human cortical explants expand cortical folds, including HAPLN1, Lumican, and Collagen 1. (C) Genetic manipulations that alter ECM connections to cortical neural progenitor cells alter survival and self-renewal properties. aRG = apical radial glia; bRG = basal radial glia; CSF = cerebral spinal fluid.

The ECM encompasses noncellular components of tissues and organs and comprises fibrous proteins (e.g., collagens, laminins) and heparan sulfate proteoglycans (HSPGs) (i.e., proteins linked to glycosaminoglycan chains) that form a mechanically supportive extracellular meshwork (Frantz and others 2010) (Figure 7A). What is often not appreciated is that the ECM is a molecularly diverse and dynamic structure, undergoing constant remodeling. The diversity of the ECM is exemplified within each of its component macromolecular classes; for instance, there are 28 different types of collagens. The ECM mediates a diverse array of functions, not only providing structural support but also modulating cell differentiation, tissue morphogenesis, and homeostatic functions (Frantz and others 2010). Cells actively adhere to the ECM through receptor-ligand interactions, involving several macromolecules, including integrin and dystroglycan receptors. Cytoskeletal coupling to the ECM regulates cellular structure, cell movements, tissue elasticity, and tensile strength. Furthermore, HSPGs in the ECM, including glypicans, perlecans, and syndecans, modulate cell signaling. For instance, mutations in glypican-1 suppress Fgf signaling (Jen and others 2009), loss of perlecan reduces Fgf and Shh signaling (Giros and others 2007), and a mutation in syndecan-1 interferes with Wnt signaling (Wang and others 2012). Given its inherent complexity, deciphering the impact of the ECM on cortical development and folding is a challenging endeavor.

Recently, single-cell transcriptomic studies have revealed the dynamic nature of ECM composition during developmental transitions in the developing cortex, suggesting that bRG may establish their own ECM-rich niche in the oSVZ (Pollen and others 2015). Indeed, ECM gene expression is most abundant in the cortical germinal zones (VZ, iSVZ, oSVZ) in both gyrencephalic and lissencephalic species (Amin and Borrell 2020). In rodents, ECM genes that are expressed at high levels in the cortical VZ are down-regulated in the SVZ and during neurogenesis (Arai and others 2011; Fietz and others 2012). A similar dynamic remodeling of the ECM is observed in the gyrencephalic ferret cortex, not only during neurogenesis but also as development proceeds (Martinez-Martinez and others 2016). Thus, the ECM composition of the neurogenic niche is dynamically altered as neurogenesis proceeds (Amin and Borrell 2020; Fietz and others 2012).

Intriguingly, the levels and complexity of ECM gene expression are much higher in human aRG and bRG compared to rodents, indicative of an even more intricate neurogenic niche (Florio and others 2015; Pollen and others 2015). Using cerebral organoids, differences in ECM gene expression have been observed in aRG derived from human or chimpanzee pluripotent stem cells (Camp and others 2015), which has been speculated to contribute to the differences in cortical expansion in these species during evolution (Amin and Borrell 2020). Finally, consistent with a potential functional link between ECM components and cortical gyration, ECM gene expression in the oSVZ varies between areas that will form sulci and those that will form gyri in the gyrencephalic ferret brain (de Juan Romero and others 2015). In support of a functional link between the ECM and cortical gyration, applying ECM components such as HAPLN1, Lumican, and Collagen 1 directly to human cortical slices could induce folding (Long and others 2018) (Figure 7B).

How might the ECM affect cortical folding? Morphologically, both apical and basal RG extend basal projections that retain a connection with the ECM-rich basal lamina (Fietz and others 2010; Hansen and others 2010; Reillo and others 2011). ECM components are also rich in the meninges, including laminins, which exert their functions by binding integrin or other receptors on aRG (Amin and Borrell 2020). There are now also several examples of how alterations to the ECM can affect cortical NPC cell division patterns, beginning from the NEC stage. For instance, mutations in several HSPGs enriched in the cortical VZ reduce NEC proliferation, leading to precocious neuronal differentiation (Amin and Borrell 2020). Furthermore, laminins, which exert their functions by binding integrin or other receptors, are expressed at high levels in the cortical germinal zones, and their overexpression can stimulate cortical NPCs to proliferate in vitro (Amin and Borrell 2020). Moreover, there is a correlation between ECM components and high levels of expression of integrin avb3 in the oSVZ of the human cortex, the knockdown of which interferes with bRG proliferation in the ferret and human brain (Fietz and others 2010; Reillo and others 2011) (Figure 7C). Conversely, blocking β1-integrin signaling leads to a detachment of aRG from the apical surface and programmed cell death (Radakovits and others 2009) (Figure 7C). Finally, knocking out laminin α2/α4 in the cortical meninges, or surgical removal of the meninges, leads to aRG rounding up and undergoing cell death (Radakovits and others 2009) (Figure 7C). Thus, by controlling tissue stiffness and cell signaling, the ECM is a central regulator of cortical NPC differentiation, neuronal migration, and subsequent cortical folding (Llinares-Benadero and Borrell 2019).

In humans, individuals with mutations in the secreted ECM protein galectin-3 binding protein (LGALS3BP), which binds to several ECM proteins, including integrins, collagens, and laminins, have altered patterns of cortical gyrification (Kyrousi and others 2021). While Lgals3bp is not expressed during mouse cortical development, LGALS3BP is expressed in human cortical aRG, bRG, and IPCs (Fietz and others 2012; Telley and others 2019), and in the ferret, Lgals3bp is expressed at higher levels in presumptive gyri versus sulci (de Juan Romero and others 2015). Strikingly, when overexpressed in the murine cortex, human LGALS3BP induces cortical folding, correlating with the mispositioning of cortical NPCs (Kyrousi and others 2021). Conversely, in LGALS3BP mutant human cerebral organoids, the bRG pool is reduced in number (Kyrousi and others 2021). Together, these data support a model in which LGALS3BP regulates the anchoring of aRG to the ECM, and in its absence, bRG cannot delaminate.

Finally, the importance of the ECM and cell adhesion in directing the formation of the radial glial scaffold is further supported by evidence from the double knockout of *Flrt1/Flrt3* adhesion molecules, which increases migration and tangential dispersion, leading to neuronal clustering and cortical folding in mice (Del Toro and others 2017). Interestingly, in these double knockouts, cortical folds exist in the absence of basal NPC expansion, indicating that there is more than one mechanism supporting cortical folding. Notably, the continuity of the radial glial scaffold is similarly perturbed in other mouse mutants that form cortical folds, including *Neurog2/Ascl1* Split-Cre “deleter” mice (Han and others 2021). Overall, these studies support the idea that a discontinuity of neuronal migration can contribute to the formation of gyri and sulci.

## Conclusions and Future Directions

Cortical folding is supported by many cellular mechanisms, the most studied being bRG pool expansion. Multiple genes have now been identified that support a link between bRG expansion and cortical folding, including intrinsic and extrinsic factors. Taken together, these findings further support the assertion that cortical folding is driven by focal hot spots of NPC proliferation, especially of bRG, which translates into sites of high and low neurogenesis. Intriguingly, many of these genes have undergone evolutionary changes to promote cortical folding. However, recent studies have implicated new mechanisms that support cortical gyrification, including alterations to the periodicity of neurogenesis across the cortical VZ and the curved structure of the radial glial scaffold. These latter two mechanisms may help to explain why primary folds form in stereotyped regions of the neocortex. Strikingly, these asymmetric patterns of proliferation and neurogenesis are predetermined by a molecular blueprint that includes several newly evolved genes.

Despite these recent findings, it remains unclear how these genes act in a coordinated fashion to select stereotyped sites of primary gyri formation, and even less is known about the regulatory networks that govern how secondary gyri are built upon the primary folds. Indeed, genes that drive cortical folding are diverse in function, ranging from cell signaling molecules (NOTCH2NL) to metabolic enzymes (TKTL1), serine protease inhibitors (SERPINA3), GTPase activating proteins (ARHGAP11B), and proteins with domains implicated in vesicular transport (TBC1D3). SERPINA3 transcriptionally regulates GLO1, a metabolic regulator that controls the translation of NOTCH1 protein and thus may regulate cortical folding via Notch signaling. However, for many of these genes, the cellular and molecular processes that are regulated remain to be deciphered. In addition to further elucidating the gene regulatory networks that set up folding patterns, investigating the role of vesicular transport, metabolism, and epigenetics, and how they have changed during gyrencephalic species evolution, are noteworthy areas to pursue moving forward.

A final level of refinement to ECM dynamics has been observed, since transcripts for ECM genes are enriched in germinal zones, while many ECM proteins, such as HALPN1 and collagen I, are only detected at elevated levels in the cortical plate (Long and others 2018). The regulated translation of proteins ensures that proteins are localized and function in discrete tissue domains and/or at precise developmental stages (Bourke and others 2023). Understanding how and why ECM gene products are only translated in the cortical plate will be an important consideration moving forward. However, it is now known that widespread remodeling of gene networks at the transcriptional, posttranscriptional, and translational levels is required for neurodevelopment (Yang and others 2014). Indeed, mRNAs for many transcription factors that promote neuronal differentiation are not translated into proteins during embryonic brain development due to sequestration by RNA-binding proteins (e.g., Pum2, Celf2, Stau2, Cpeb4) located in cytoplasmic organelles called processing bodies, which block neurogenesis (MacPherson and others 2021; Yang and others 2014). The importance of these RNA-binding proteins in regulating the translational repression of ECM components during remodeling of the neurogenic niche will be an important area of investigation moving forward. SynTRAP, which has been used to identify ribosome-bound mRNA in cortical neurons, specifically within neurites, could be used to identify genes with local translation (Ouwenga and others 2017). A similar study could focus on NPCs at various developmental stages to uncover interesting ECM candidates.
